# New STAM, new horizon

**DOI:** 10.1080/14686996.2016.1139673

**Published:** 2016-02-12

**Authors:** Shu Yamaguchi

**Affiliations:** ^a^ Materials Engineering, University of Tokyo

I would like to celebrate the new year of 2016 together with all who have contributed to our journal, *Science and Technology of Advanced Materials* (STAM). This is a historic moment for the journal, which, as of January 2016, has a new publishing partner (Taylor and Francis Group), a reinforced editorial team and a new Editor-in-Chief.

As described on the STAM web page (http://e-materials.net/stam/purpose/), STAM was born with the objective of serving the ever-growing materials science community as one of the leading journals, covering a wide spectrum of topics. The ceaseless efforts by the former Editors-in-Chief and editorial board members has brought steady growth and development in journal visibility, in the aspects of authorship, readership, and publication, making STAM a leading Gold Open Access journal in the materials science field. STAM is currently supported jointly by the National Institute of Materials Science (NIMS) and the Swiss Federal Research Institute for Materials Science and Engineering (Empa). STAM has become a unique scientific journal owing to its non-commercial nature and the fact that it is not society based, in addition to its well-established status in Gold Open Access publishing since 2008 as a pioneer journal in materials science.

The new team will add a few new flavors to STAM, while reserving its core editorial policy and philosophy. Here, as a new Editor-in-Chief, I’d like to introduce new ideas for the STAM editorials this year, which will begin with *new endeavor* to a frontier of *new horizon*: *new STAM, new horizon*

STAM covers a wide spectrum of materials science and engineering (materials science hereafter) and accepts only articles that report high quality scientific content. The field of materials science is rapidly expanding through fusion with related scientific areas, increasing further its multidisciplinary impact. No-one doubts that materials science is a key to the future sustainable society. As clearly stated by Prof. Kitazawa, “A new materials science is born, when a novel material is found.” We are always keen for the birth of new “materials science” and glad if STAM can contribute to the materials science community by finding an unpolished gem from the mass of submitted research reports. We welcome any articles of the *first message* to novel and epoch-making topics, while paying careful attention to the quality of supporting data and evidence.

I and my colleagues from STAM Editorial Board are hoping to contribute to the community by continuously publishing high quality *Focus Issues* on various topics, in addition to *Review* articles on contemporary topics in materials science, as in the previous volumes. STAM Focus Issues include original and review articles, which address a hot topic in any area of materials research, from fundamental science to applications. Most of those articles are invited papers, but we will try to complement them with general contributions. We will also encourage submission of review articles covering a broader spectrum. We will soon start a new article category called “*Vision*,” which will be commissioned articles written by renowned scientists. Rather than being simple review articles, which normally summarize scientific topics on the basis of “common understandings” or concepts generally accepted by the specialists in the corresponding area, *Vision* articles will be more personal *future prospects* of specific areas, containing authors’ *opinions*. The editorial board will also carry out surveys for the possible inclusion of short articles.

In order to increase the visibility of STAM, we are planning to initiate membership schemes. Scientific publishing is dynamically changing, owing to the rapid growth of science and technology. Open Access publication is receiving increasing attention from scientists because of the shift of funding policies to Open Access publication for projects supported by tax-payers’ funds, as can be found in Japan via Japan Society for the Promotion of Science (JSPS) [1]. One good example of such Open Access publications was realized in STAM by a comprehensive review [2] of Prof. Hosono’s group as an outcome of a survey of new superconducting materials funded through the FIRST program supported by the Japanese Government. The review paper, 88 pages in length, which is possible due to an electronic journal with no page limit, reports almost 1000 materials, regardless of the emergence of superconductivity. We believe that the review had enormous impact on superconductivity materials science, as anyone can access the vast scientific achievement to extract further benefit, utilizing most of the Open Access publication. In order to support scientists who are in this rapid stream of Open Access publishing in the field of materials science, not only can STAM serve as an alternative journal to both the commercial journals and the society journals, but it also can provide the best quality editing and publishing services with Taylor & Francis. We are very much looking forward to close communication with the STAM audience, to providing quality services, and to sharing the benefits of Open Access publishing in materials science together.

Again, I wish you all a happy 2016, the year of *new STAM* for *new horizon*.

Shu Yamaguchi STAM Editor-in-Chief *Materials Engineering, University of Tokyo*
